# An environmentally friendly and productive process for bioethanol production from potato waste

**DOI:** 10.1186/s13068-016-0464-7

**Published:** 2016-03-02

**Authors:** Fangzhong Wang, Yi Jiang, Wei Guo, Kangle Niu, Ruiqing Zhang, Shaoli Hou, Mingyu Wang, Yong Yi, Changxiong Zhu, Chunjiang Jia, Xu Fang

**Affiliations:** State Key Laboratory of Microbial Technology, School of Life Science, Shandong University, Jinan, Shandong China; School of Chemistry and Chemical Engineering, Shandong University, Jinan, Shandong China; Shandong Bio Sunkeen Co., Ltd, Jining, Shandong China

**Keywords:** Sweet potato residues, Cellulase, Pectinase, Bioethanol, Viscosity reduction, Glucose production

## Abstract

**Background:**

China is the largest sweet potato producer and exporter in the world. Sweet potato residues (SPRs) separated after extracting starch account for more than 10 % of the total dry matter of sweet potatoes. In China, more than 2 million tons of SPRs cannot be utilized, and the unutilized SPRs are perishable and result in environmental pollution. Thus, an environmentally friendly and highly efficient process for bioethanol production from SPRs should be developed.

**Results:**

The swelling behaviour of cellulose causes high-gravity sweet potato residues to be recalcitrant to enzymatic hydrolysis. Cellulase plays a major role in viscosity reduction and glucose production. In contrast, pectinase has a minor role in viscosity reduction but acts as a “helper protein” to assist cellulase in liberating glucose, especially at low cellulase activity levels. In total, 153.46 and 168.13 g/L glucose were produced from high-gravity SPRs with cellulase and a mixture of cellulase and pectinase, respectively. These hydrolysates were fermented to form 73.37 and 79.00 g/L ethanol, respectively. Each kilogram of dry SPR was converted to form 209.62 and 225.71 g of ethanol, respectively.

**Conclusion:**

The processes described in this study have an enormous potential for industrial production of bioethanol because they are environmentally friendly, highly productive, economic with low cost, and can be easily manipulated.

**Electronic supplementary material:**

The online version of this article (doi:10.1186/s13068-016-0464-7) contains supplementary material, which is available to authorized users.

## Background

The extensive use of fossil fuels has stimulated economic development and has promoted the progress of human civilization since the twentieth century. However, problems involving the rapid depletion of fossil fuels have caused serious concern. The incremental demand for an energy supply has led to a rapid reduction in fossil fuels and the subsequent world energy crisis [1]. The greenhouse effect caused by the massive combustion of fossil fuels has gradually destabilized the world ecosystem and human social systems [2]. Consequently, the need for sustainable and environmentally friendly alternative energies has been recognized by society [1, 3]. Because of its unique advantages over other renewable energies, bioethanol produced from agricultural or industrial waste has received special attention. The use of renewable and carbon neutral bioethanol is considered an effective strategy to counter the greenhouse effect [4]. The chemical intermediates that accompany bioethanol production can be produced by biorefineries [5], and bioethanol is compatible with the current supply of liquid fuels [6]. Therefore, bioethanol is one of the most promising alternatives to fossil fuels that can ensure energy security and address environmental pollution problems [7, 8]. Many new technologies for bioethanol production from various wastes are being extensively studied, while environmentally friendly processes are being constantly improved [9–13].

China is the largest sweet potato producer and exporter in the world [14], with more than 71 million tons of sweet potatoes being produced each year. Sweet potatoes are one of the important raw materials for starch preparation. Sweet potato residues (SPRs) separated after extracting starch account for more than 10 % of the total dry matter of sweet potatoes. In China, more than 2 million tons of SPRs cannot be utilized, perhaps owing to their high viscosity. Moreover, the unutilized SPRs are perishable, and release methane as they turn rancid because they contain abundant polysaccharides and proteins [15–18]. The influence of methane, the second-most common greenhouse gas, on climate change is more than 25 times greater than that of carbon dioxide over a 100-year period [19]. Therefore, unutilized SPRs pose a serious problem of environmental pollution. Recently, acid-catalysed hydrolysis methods of releasing sugar from potato wastes were reported for bioethanol production [20, 21]. However, these processes increase the discharge of industrial waste water [22] and raise costs because they require investment in corrosion-resistant equipment and production of fermentation inhibitors [1]. Thus, these technologies have limited industrial application. Therefore, an efficient and environmentally friendly method that uses enzymatic hydrolysis should be developed for bioethanol production from potato waste.

Enzyme-catalysed hydrolysis usually occurs in lower substrate contents owing to the high viscosity of potato waste, leading to poor efficiency and high energy consumption [23, 24]. Energy consumption is a major part of the cost of ethanol production [25]. High-gravity fermentation technology could produce very high ethanol titres, which could decrease energy consumption for ethanol distillation and waste distillation treatment [26, 27]. Therefore, bioethanol production based on corn and wheat mashes has received significant attention [28, 29]. However, the feasibility of bioethanol production based on highly concentrated SPRs has rarely been reported.

In the present study, the feasibility of converting high-gravity SPRs into bioethanol was studied. The performance of different enzyme systems during high-gravity SPRs hydrolysis was investigated. Then, the possible roles of cellulase and pectinase during enzymatic hydrolysis of high-gravity SPRs were investigated. Finally, 79.00 g/L ethanol was produced from enzymatic hydrolysates by *Saccharomyces cerevisiae*. Our findings may be used as a reference for industrial production of bioethanol based on SPRs.

## Results and discussion

### Chemical component analysis of SPRs

SPRs are starch-containing industrial waste. Their chemical composition was investigated to evaluate their potential for SPR-based bioethanol production. Dry SPR is composed of 30.01 ± 1.27 % (w/w, dry weight) starch, 29.29 ± 3.17 % cellulose, 13.79 ± 1.12 % pectin, 4.16 ± 0.50 % lignin and 1.98 ± 1.58 % ash. Each kilogram of dry SPRs can release 658.96 ± 41.74 g glucose, 178.34 ± 14.50 g galacturonic acid, 80.28 ± 3.66 g galactose and 10.07 ± 1.83 g xylose according to the National Renewable Energy Laboratory protocols (see “Methods” section). It is obvious that the hemicellulose content is lower in SPRs.

**Fig. 1:**
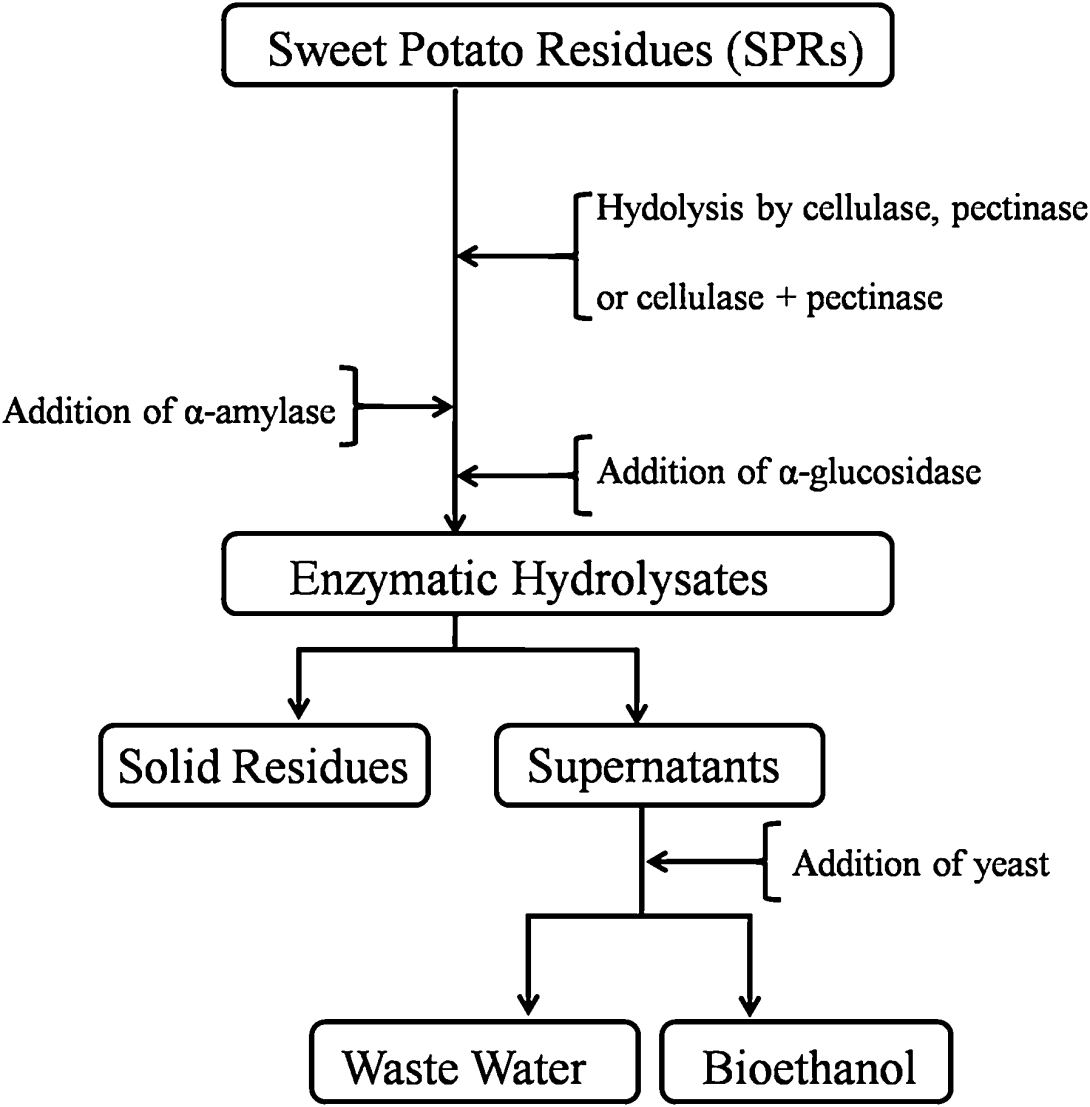
A scheme of over-process of bioethanol production from high-gravity SPRs

Meyer et al. [30] reported that galactose substitutes for side chains of galacturonan, which is the backbone of pectin in dicotyledonous plants. Sweet potato is a type of dicotyledonous plant. The contents of galactose and galacturonic acid were relatively high in the SPRs, which may suggest that there is plenty of pectin in the SPRs. The pectin is composed of galacturonan with highly branched galactose. Starch comprises 64.81 ± 1.59 % of sweet potato, and the total content of cellulose and pectin is less than 13 %. Therefore, more than half of the starch is extracted by the starch-processing industry, and the contents of cellulose and pectin are apparently increased in the SPRs. Cellulose and pectin are crosslinked polymers that can exhibit swelling behaviour when exposed to solvent agents [31–34]. It is speculated that cellulose and pectin in SPRs could form a complex network to limit the mobility of enzyme molecules. Furthermore, the viscosity of SPRs becomes higher as substrate concentration increases, leading to the lower mobility of enzyme. Therefore, the contents of cellulose and pectin have a great influence on bioethanol production. The impacts of lignin and ash on enzyme hydrolysis and ethanol production can be neglected owing to their low contents. After starch was partially extracted from sweet potato, 658.96 g of glucose was still released from each kg of SPRs. These results suggest that SPRs are potential raw material sources for glucose and bioethanol production. A scheme of over-process for glucose or bioethanol production from SPRs was proposed in Fig. 1.

### Assessment of the hydrolysis performance of different enzyme systems on high-gravity SPRs

The high-gravity SPRs were directly hydrolysed by α-amylase and α-glucosidase, but their viscosity was sharply increased, and the amount of released glucose was relatively low.

**Fig. 2:**
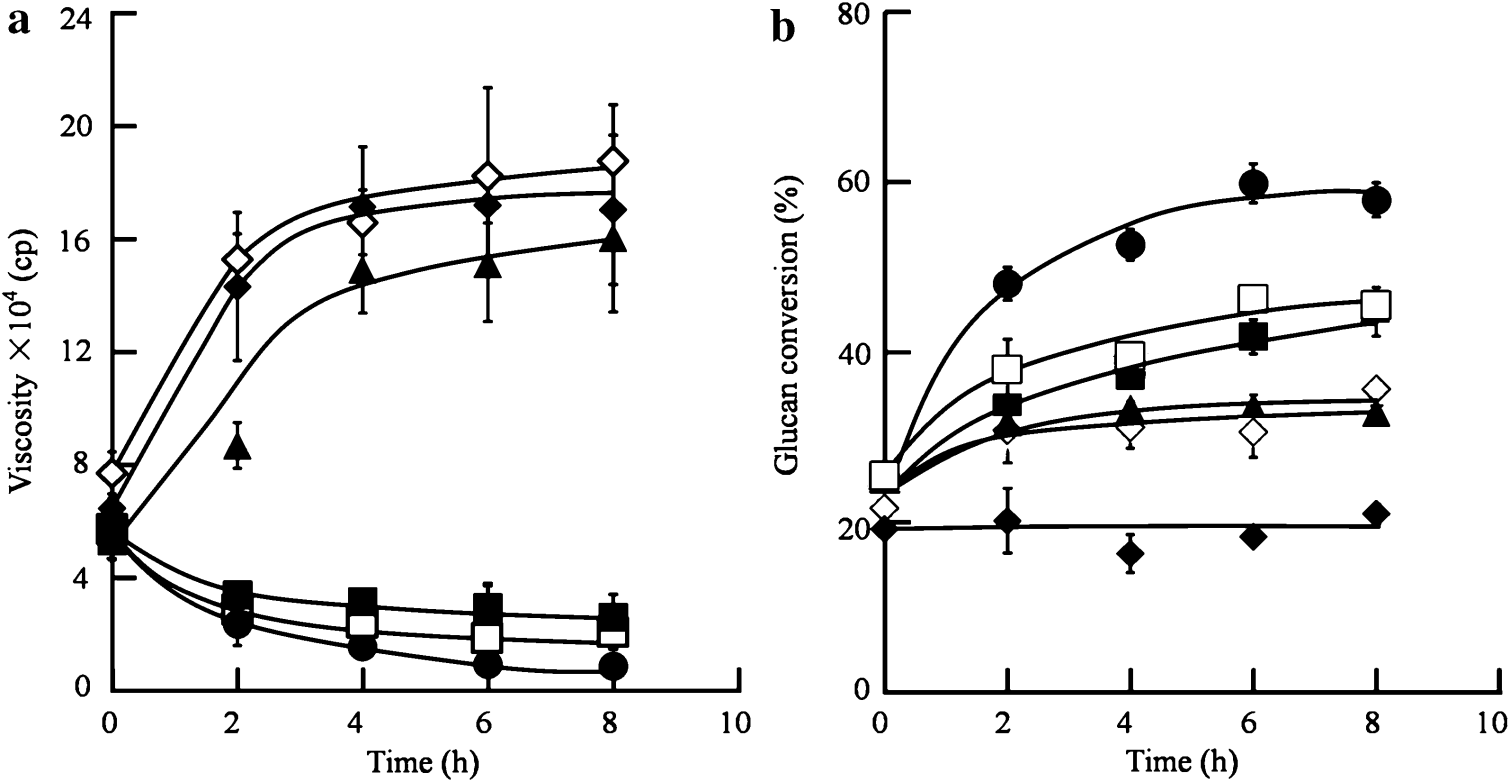
The viscosity changes (**a**) and the glucan conversions of high-gravity SPRs (**b**) during enzymatic hydrolysis. All the reactions were performed at 45 °C and 200 rpm for 8 h with an initial pH of 4.8. The concentration of SPRs in reaction system was 36 % (w/v). Three independent replicates were performed. *Solid diamonds* represent the control, *hollow diamonds* represent commercial pectinase, *solid triangles* represent enzymes derived from *A*. *niger*, *solid squares* represent *T*. *reesei* TX, *hollow squares* represent *T*. *reesei* T1, and *solid circles* represent *P*. *oxalicum* JUA10-1

To develop an efficient and environmentally friendly process, we investigated the Filter paperase (FPase), pectinase and α-amylase activities of various enzyme systems, which have the potential to degrade three SPR components: starch, cellulose and pectin. The *Penicillium oxalicum* JUA10-1, *Trichoderma reesei* T1 and *T. reesei* TX enzyme systems exhibited higher specific cellulase activities and lower specific pectinase and α-amylase activities than enzymes derived from commercial pectinase and *Aspergillus niger* (Table 1). The hydrolysis efficiencies of these enzymes on high-gravity SPRs were evaluated for two parameters, namely viscosity change and glucose production. As shown in Fig. 2a, the viscosities of SPRs without or mixed with pectinase or enzymes (10 mg of soluble protein/g dry SPRs) derived from *A. niger* were increased, while those of the *P. oxalicum* JUA10-1, *T. reesei* T1 and *T. reesei* TX enzyme systems were considerably reduced. Furthermore, the glucan conversions of the *P. oxalicum* JUA10-1, *T. reesei* T1 and *T. reesei* TX enzyme systems were higher than those of pectinase or *A. niger* (Fig. 2b). Therefore, there may be a correlation between the amount of released glucose and the viscosity reduction of a reaction system. It is reported that cellulose fibre diameter could increase sevenfold in amine oxide/water systems [35], and the reconstituted cellulose could adsorb 338 mL water per 100 g of sample [36]. In combination with the enzymatic activities shown in Table 1, these results suggest that the high viscosity of high-gravity SPRs results from cellulose swelling, not starch and pectin. One possible reason for higher glucose production is that the viscosity reduction of a reaction system leads to improved mobility of α-amylase and α-glucosidase, releasing more glucose from starch, not just from cellulose hydrolysis.

**Table 1:** The comparisons of the specific activities of enzymes derived from different enzymatic systems

Specific activity (U/mg)	FPase	Pectinase	α-amylase
*P. oxalicum* JUA10-1	0.60 ± 0.02	13.97 ± 1.33	0.04 ± 0.01
*T. reesei* T1	0.41 ± 0.02	1.23 ± 0.02	0.02 ± 0.00
Commercial pectinase	0.01 ± 0.00	529.79 ± 36.01	0.07 ± 0.00
*A. niger*	0.05 ± 0.00	2.12 ± 0.12	0.07 ± 0.01
*T. reesei* TX	0.28 ± 0.02	0.81 ± 0.02	0.01 ± 0.00

### The roles of cellulase and pectinase during enzymatic hydrolysis of high-gravity SPRs

**Fig. 3:**
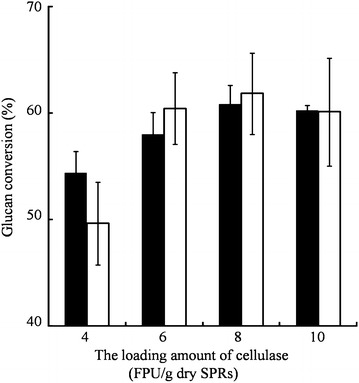
Glucose released from high-gravity SPRs by *T*. *reesei* TX and *P*. *oxalicum* JUA10-1. All the reactions were performed at 45 °C and 200 rpm for 6 h with an initial pH of 4.8. The concentration of SPRs in reaction system was 36 % (w/v). Three independent replicates were carried out. *Solid bars* represent *T*. *reesei* TX, and *hollow bars* represent *P*. *oxalicum* JUA10-1

As shown in Fig. 2b, glucan conversion of *P*. *oxalicum* JUA10-1 is the highest. The specific FPase and pectinase activities in *P. oxalicum* JUA10-1 were much higher than those in *T. reesei* T1 or *T. reesei* TX enzyme systems (Table 1). However, we were interested to determine which enzymes are mainly responsible for glucose release. Therefore, equal amounts of FPase activity were added to the reactions containing high-gravity SPRs, after which it was found that there was no significant difference between the glucan conversions of *T. reesei* TX and *P. oxalicum* JUA10-1 (Fig. 3), although the specific pectinase activity of *P. oxalicum* JUA10-1 was 17-fold higher than that of *T. reesei* TX. The glucan conversion reached 62.37 %, and the glucose concentration in the reaction system was 153.46 g/L (Table 2). Thus, it was suggested that FPase activity greatly contributes to the liberation of glucose from SPRs.

**Table 2:** Comparison of different processes using potato waste materials to produce ethanol

Raw materials	Substrate concentration (w/v)	Pretreatment method	Glucose concentration (g/L)	Ethanol concentration (g/L)	Glucan conversion (%)	Glucose to ethanol conversion (%)	Ethanol yield (g/g, dry weight)	Reference
Sweet potato residues	36 %	Enzymatic hydrolysis^a^	168.13 ± 2.62	79.00 ± 0.87	68.34 ± 1.07	67.16 ± 0.74	0.23 ± 0.00	This study
Sweet potato residues	36 %	Enzymatic hydrolysis^b^	153.46 ± 2.01	73.37 ± 1.87	62.37 ± 0.82	62.37 ± 1.59	0.21 ± 0.01	This study
Potato pulp	30 %	Hydrothermal pretreatment and enzymatic pretreatment^c^	114	Less than 60	68	Less than 68	Less than 0.2	[48]
Potato peel waste	2 %	Enzymatic hydrolysis^d^	18.48 ± 0.65	7.50 ± 0.28	–	–	–	[23]
Waste potato mash	–	Physical method^e^	Less than 80	35	–	–	–	[24]
Potato peel + substandard mash	1:1	Enzymatic hydrolysis^f^	–	48.6 ± 1.3	–	42.5	0.19	[57]
Potato tuber mash	1:1	Acid-catalysed hydrolysis^g^	100	32.9	–	–	–	[20]
Potato peel waste	4 %	Acid-catalysed hydrolysis^h^	18.15	6.97	–	–	–	[23]
Potato starch residue stream	–	Acid-catalysed hydrolysis^i^	18.9	5.62 ± 0.21	–	–	–	[21]

^a^4 FPU/g dry SPRs + 1000 PGU/g dry SPRs, 45 °C for 6 h, cellulase from *T*. *reesei* TX, pectinase was generously provided by Qingdao Vland Biotech Inc

^b^6 FPU/g dry SPRs, 45 °C for 6 h, cellulase from *T*. *reesei* TX

^c^Hot water pretreatment, 121 °C for 20 min, 5 FPU/g dry weight, 50 °C for 48 h, enzyme from *A. cellulolyticus*

^d^0.12 U/g dry weight at 85 °C for 1 h, enzyme from Termamyl 120 L, then 12 U/g dry weight at 44 °C for 2.5 h, enzyme from Viscozyme, and finally, 1 g Celluclast per g dry weight at 50 °C for 2 h

^e^Agitation at 120 rpm for 3 h at a temperature chosen by the design based on a preliminary study

^f^The amount of enzyme, consisting of α-amylase + Pectinase + Enzyme complex, is 0.1 %. They were incubated at 50 °C for 21 h

^g^60 min in 1 M HCl at 100 °C

^h^0.5 M HCl, 121 °C for 15 min

^i^60 min in 1 % H_2_SO_4_ at 100 °C

As shown in Fig. 3, the amount of released glucose was not increased as the activities of cellulase derived from the *P. oxalicum* JUA10-1 or *T. reesei* TX enzyme systems exceeded 6 FPU/g dry SPRs. One possible reason is that the pectinase activity of the enzyme system derived from *P. oxalicum* JUA10-1 or *T. reesei* TX is too low to adequately degrade pectin that still limits cellulase accessibility to cellulose. Therefore, the role of pectinase should be explored for further improvement of glucan conversion. The enzyme solutions derived from *T. reesei* TX with commercial pectinase were chosen for further study because the enzyme system of *T. reesei* TX has the low specific pectinase activity (Table 1).

**Fig. 4:**
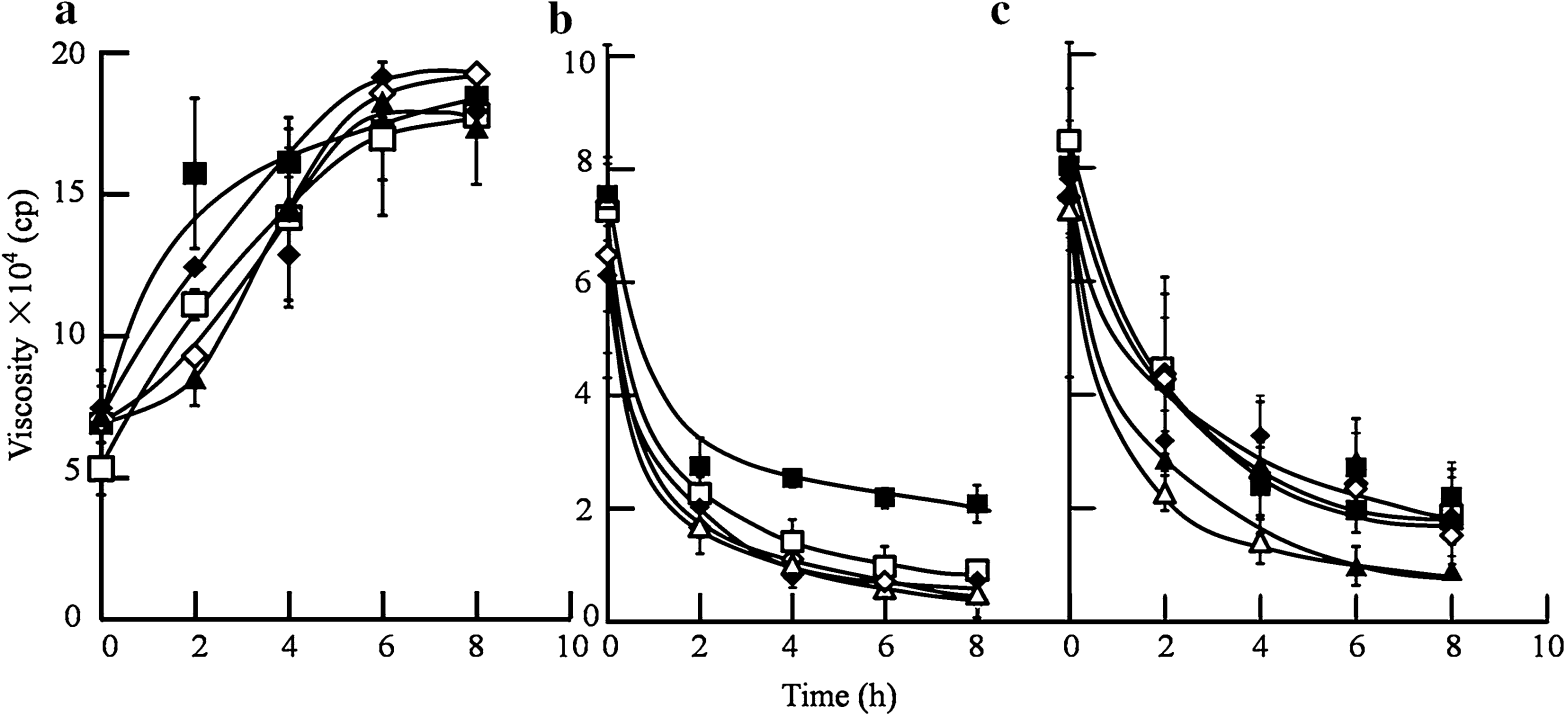
Viscosity change of high-gravity SPRs when pectinase (**a**), cellulase (**b**) and mixtures (**c**) were used. All the reactions were performed at 45 °C and 200 rpm for 8 h with an initial pH of 4.8. The concentration of SPRs in reaction system was 36 % (w/v). Three independent replicates were carried out. **a** *Solid squares* represent the control, *hollow squares* represent 500 PGU/g dry SPRs, *solid diamonds* represent 1000 PGU/g dry SPRs, *hollow diamonds* represent 5000 PGU/g dry SPRs and *solid triangles* represent 10,000 PGU/g dry SPRs. **b** *Solid squares* represent 2 FPU/g dry SPRs, *hollow squares* represent 4 FPU/g dry SPRs, *solid diamonds* represent 6 FPU/g dry SPRs, *hollow diamonds* represent 8 FPU/g dry SPRs, and *hollow triangles* represent 10 FPU/g dry SPRs. **c** *Solid squares* represent 2 FPU/g dry SPRs; *hollow squares* represent 2 FPU/g dry SPRs + 500 PGU/g dry SPRs; *solid diamonds* represent 2 FPU/g dry SPRs + 1000 PGU/g dry SPRs; *hollow diamonds* represent 2 FPU/g dry SPRs + 2500 PGU/g dry SPRs; *solid triangles* represent 2 FPU/g dry SPRs + 5000 PGU/g dry SPRs; and *hollow triangles* represent 4 FPU/g dry SPRs

The viscosity was dramatically increased when commercial pectinase was used, even with the addition of 10,000 PGU/g dry SPRs (Fig. 4a). In contrast, the viscosity was decreased when the activity of cellulase derived from *T. reesei* TX was more than 4 FPU/g dry SPRs in the reaction system (Fig. 4b). These results suggest that cellulase, but not pectinase, plays a major role in viscosity reduction. As shown in Fig. 4c, there is no significant change when pectinase is mixed into the reaction system. The rates and degrees of viscosity reduction were lower than those of 4 FPU/g dry SPRs. Thus, there may be no synergistic effect between cellulase and pectinase on viscosity reduction.

These findings seem to be in conflict with reports on viscosity reduction of high-gravity potato mash [37]. Celluclast 1.5 L contributed little to viscosity reduction, but pectin-degrading enzymatic solutions such as Pectinex Ultra SP-L or Viscozyme L could effectively decrease the viscosity of high-gravity potato mash [37]. However, there are also reports that cellulase performed better than pectinase in viscosity reduction of high concentrations of raw materials containing cellulose and pectin [38–40]. The results in this study support the latter viewpoint. The swelling behaviours of starch, cellulose and pectin depend on the ratio of crystalline and amorphous regions and reaction conditions [34, 36, 41–43]. Therefore, the conflicting results may be resulting from the ratio of cellulose and pectin, the structural disparity of cellulose and pectin, the conditions for viscosity reduction, etc.

**Fig. 5:**
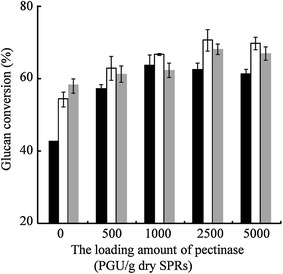
Glucose released from high-gravity SPRs when the mixtures of cellulase + pectinase were used. All the reactions were performed at 45 °C and 200 rpm for 6 h with an initial pH of 4.8. The concentration of SPRs in reaction system was 36 % (w/v). Three independent replicates were carried out. *Black bars* represent 2 FPU/g dry SPRs with various PGU/g dry SPRs, *hollow bars* represent 4 FPU/g dry SPRs with various PGU/g dry SPRs, and *grey bars* represent 6 FPU/g dry SPRs with various PGU/g dry SPRs

There was no significant change (p > 0.05, n = 3) on the glucan conversion without or with addition of pectinase (Additional file 1). It was suggested that pectinase plays a minor role in the glucan conversion of SPRs. When the loading amount of cellulase was more than 4 FPU/g dry SPRs, the viscosity of the reaction system was considerably reduced, and the glucan conversion was significantly improved (p < 0.05, n = 3) (Figs. 4c, 5). It was suggested that cellulase plays a major role in degrading SPRs. The glucan conversion of cellulase mixed with commercial pectinase was significantly higher than that with only the addition of 2 FPU/g dry SPRs (p < 0.05, n = 3). Furthermore, the amount of released glucose was comparable to that achieved with the addition of 6 FPU/g dry SPRs at a considerably reduced total protein load. When the enzyme of 2 FPU/g dry SPRs + 1000 PGU/g dry SPRs was loaded, the total amount of protein loading (50.2 mg) was nearly half of the amount achieved when using the 6 FPU/g dry SPRs (115.7 mg). Therefore, the total amount of protein loading was reduced approximately twofold. The addition of cellulase mixed with pectinase could significantly improve glucan conversion more than that achieved with only the addition of 4 FPU/g dry SPRs (p < 0.05, n = 3). The glucan conversion of cellulase (4 FPU/g dry SPRs) mixed with the pectinase (above 1000 PGU/g dry SPRs) was significantly higher than that achieved when using only 6 FPU/g dry SPRs (p < 0.05, n = 3). Moreover, glucan conversion was enhanced when cellulase (6 FPU/g dry SPRs) mixed with pectinase solutions was used. The addition of commercial pectinase into low concentration of cellulase solutions could greatly improve glucan conversions. Pectinase could clear the barriers, such as pectin, which intertwine cellulose to improve cellulase motion on cellulose fibres. Therefore, pectinase acts as a “helper protein” to assist cellulase to degrade cellulose during the hydrolysis of high-gravity sweet potato residues, especially at low cellulase activity levels.

As mentioned above, the synergistic action of cellulase and pectinase was observed, and pectinase is a “helper protein” during high-gravity sweet potato residues hydrolysis. Therefore, the optimum concentration of pectinase in the mixture enzyme system depends on the loading amount of cellulase.

It was reported that cellulase contributes more to starch liberation than pectinase during cassava pulp hydrolysis [44]. The concerted action of cellulolytic enzymes and pectinase was observed when pretreated corn stover was hydrolysed, and their mixture reduced half of the loading amount of soluble protein [45]. These reports are consistent with the results in the present study. There are many reports on the synergistic effects of cellulase and accessory proteins on the stimulation of lignocellulolytic hydrolysis. For example, xylanase and lytic polysaccharide monooxygenase can enhance the hydrolysis efficiency of cellulase on high-gravity steam-pretreated poplar and corn stover [46]. GH10 endo-xylanases and GH5 xyloglucanases had strong concerted effects with cellulase on the hydrolysis of pretreated lignocellulosic substrates, but the synergistic effects exhibited substrate dependence [47]. However, when 6 FPU/g dry SPRs was added, there was no significant synergistic effect of cellulase and pectinase on glucan conversion. The possible reason is as follows. Pectin and cellulose intertwine starch to restrict α-amylase accessible in SPRs. When the concentration of cellulase is lower, pectinase could clear the barrier, such as pectin, to improve cellulase motion on cellulose fibres. Thus, glucan conversion is improved, and the synergistic action of cellulase and pectinase can be observed. However, more cellulose-degrading sites in SPRs are occupied by cellulase molecules with the increasing amount of cellulase loading. The complex network is quickly broken without further improvement in the motor ability of cellulase. Therefore, the concerted effect between cellulase and pectinase may not be obvious.

### Ethanol production from enzymatic hydrolysates by *S. cerevisiae*

**Fig. 6:**
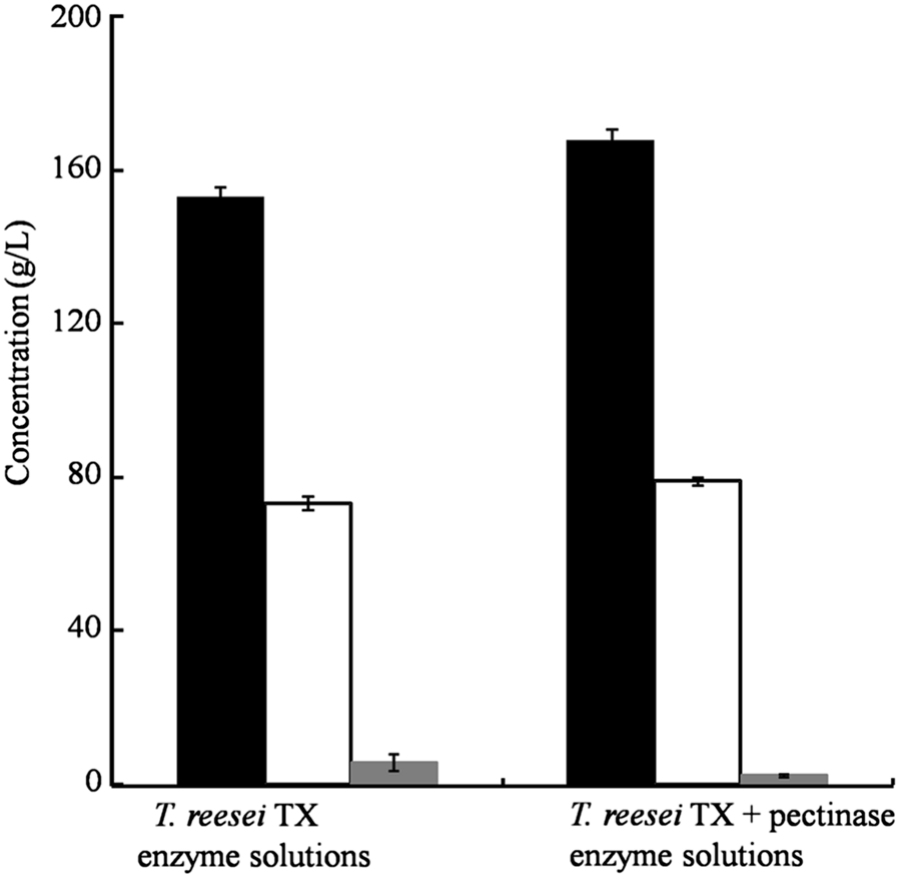
Ethanol production from enzymatic hydrolysates by *S. cerevisiae*. The initial *S. cerevisiae* inoculation amount was 0.5 %. All the reactions were performed at 30 °C for 72 h. Three independent replicates were performed. *Black bars* represent glucose concentrations after enzymatic hydrolysis, *hollow bars* represent ethanol concentrations after fermentation by *S. cerevisiae*, and *grey bars* represent the remaining glucose concentrations after fermentation by *S. cerevisiae*

It is reported that the enzymatic hydrolysates of potato pulp could be fermented into ethanol without addition of any nitrogen source for improving efficiency [48]. Therefore, ethanol fermentations were directly performed with SPRs hydrolysed by *T*. *reesei* TX or *T*. *reesei* TX+ pectinase enzymatic solutions without supplementing any source. The results are shown in Fig. 6. The glucose concentration with the enzyme from *T*. *reesei* TX was 153.46 g/L after enzymatic hydrolysis, and the ethanol concentration reached 73.37 g/L. In addition, 5.76 g/L of glucose was left after 72-h fermentation by *S. cerevisiae*. When the enzyme of *T*. *reesei* TX+ pectinase was added, the glucose concentration was 168.13 g/L after enzymatic hydrolysis, ethanol was 79.00 g/L and 2.46 g/L of glucose remained. Therefore, almost the entire glucose was converted into ethanol without supplementing any nitrogen source.

### Comparison of different processes using potato waste materials to produce ethanol

Table 2 contains a comparison of bioethanol production from potato waste materials by different processes reported in the literature. Although the acid-catalysed methods have relatively higher conversions [23], the glucose or ethanol concentrations are relatively lower. The drawbacks, such as fermentation inhibitor production, potential environmental pollution problems associated with waste disposal and the high cost of special equipment, are obvious [1, 49]. Of the results listed in Table 2, the glucose and ethanol concentrations in this study are the highest among enzyme-catalysed methods. Furthermore, the processes are easily manipulated and are of low cost because they do not require further pretreatment or the addition of other enzymes, which react under different conditions, to produce ethanol. In addition, the amount of wastewater in these processes may be relatively lower among enzyme-catalysed methods because of high-gravity substrates. Therefore, they are eco-friendly, highly efficient, of low cost, and easily manipulated processes for bioethanol production.

## Conclusion

The present results showed that 79.00 g/L of ethanol could be obtained from high-gravity SPRs using enzyme hydrolysis. The swelling behaviour of cellulose was believed to cause high-gravity SPRs to be recalcitrant to enzymatic hydrolysis. Cellulase plays major roles in viscosity reduction and glucose production. Pectinase seems to contribute little to viscosity reduction but acts as a “helper protein” to assist cellulase in liberating glucose, especially at low cellulase activity levels. The concentrations of glucose and ethanol produced from potato wastes were the highest in this study. Compared with other processes, the processes described here have an enormous potential for industrial-scale production of bioethanol because they are environmentally friendly, highly productive, of low cost, and easily manipulated.

## Methods

### Materials

SPRs and high sugar tolerant instant dry yeast were provided by Shandong Bio Sunkeen Co., Ltd, Jining, Shandong, China. The cellulase solutions were produced by *P. oxalicum* JUA10-1, *T. reesei* T1, *T. reesei* TX and *Aspergillusniger*, which are laboratory-maintained filamentous fungi [6]. *T. reesei* TX was obtained as follows: the *xylanase* III gene (Genbank accession number: BAA89465.2) was replaced by β-glucosidase 1 (*bgl* 1, Genbank accession number: KJ739789.1) derived from *A*. *niger* under the control of the *T. reesei* T1 *xylanase* III promoter. The methods for cellulase production were as described else [11]. The commercial pectinase solution was generously provided by Qingdao Vland Biotech, Qingdao, Shandong, China. The α-amylase (Novozymes (China) Biotechnology Co., Ltd) and α-glucosidase (Shandong Longda Bio-products Co., Ltd) were generously provided by Jiang Su Lianhai Biological Technology Co., Ltd. Citric pectin, polygalacturonic acid, soluble starch, and *p*-nitrophenyl-α-_D_-glucopyranoside were purchased from Sigma-Aldrich, St. Louis, MO, USA. Whatman No. 1 filter paper was purchased from Hangzhou Whatman-Xinhua Filter Paper, Hangzhou, Zhejiang, China. All other chemicals were bought from Sinopharm Chemical Reagent, Shanghai, China.

### Analytical methods

FPase, pectinase, α-amylase, and α-glucosidase activities were measured as described elsewhere [50–52]. The protein concentration was assayed using the Lowry method [53]. For composition analysis of SPRs, glucose, xylose and galactose were produced according to the National Renewable Energy Laboratory protocols [54]. The content of glucose was determined as described elsewhere [6]. The contents of xylose and galactose were determined using a Dionex ICS 2500 system (Thermo Scientific, Waltham, MA, USA) with a CarboPac™ PA1 analytical column (2 × 250 mm). The elution solution was a mixture of water and 400 mM NaOH at a volume ratio of 95:5. The production and measurement of galacturonic acid followed the protocols as previously described [55]. The pectin content was based on the released galacturonic acid. Ash and lignin were measured as described elsewhere [56]. Starch content was determined with the method described previously [48]. The cellulose content was determined by subtracting the starch content from the glucan content. Three independent replicates were performed.

### Viscosity measurement of SPRs during enzymatic hydrolysis

Cellulase from *P. oxalicum* A10-1, *T*. *reesei* T1, *T*. *reesei* TX, commercial pectinase and their mixtures were added to SPRs in amounts corresponding to 10 mg of soluble protein/g dry SPRs, and the viscosity change of these solutions was measured. The final SPR concentrations were adjusted to 36 % by supplementing citric acid buffer (10 mM, pH 4.8) in all viscosity tested assays. All the tubes were incubated in an oscillating machine (Type HYG-C, Suzhou Peiying Laboratory Equipment, Jiangsu, China) at 45 °C and 200 rpm for 8 h. The viscosity change of SPRs was monitored every 2 h using a viscometer (Type LVDV-C, Brookfield Engineering Labs, Middleboro, MA, USA) equipped with an s64 rotor at a speed of 3 rpm. Three independent replicates were performed.

### Determination of glucose release from SPRs during enzymatic hydrolysis

Cellulase from *P. oxalicum* A10-1, *T*. *reesei* T1, *T*. *reesei* TX, commercial pectinase and their mixtures were added to SPRs in amounts corresponding to 10 mg soluble protein/g dry SPRs, and the glucose released from the SPRs was measured. The final substrate SPRs were adjusted to 36 % by supplementing citric acid buffer (10 mM, pH 4.8) in all glucose production assays. All the tubes were incubated in an oscillating machine at 45 °C and 200 rpm for 8 h. Subsequently, 53 units of α-amylase were added and incubated at 90 °C for 2 h, and the reactions were cooled to 60 °C with the addition of 34 units of α-glucosidase. The reaction systems were incubated for 6 h to obtain the maximum glucose release. The released glucose was measured by HPLC. The HPLC system (Hitachi, Tokyo, Japan) equipped with an Aminex HPX-87H column (7.8 × 300 mm, 9 µm particle size, Bio-Rad Laboratories, Hercules, CA, USA) and an RI detector (Model L-2490, Hitachi, Tokyo, Japan) was used for glucose measurement. Separation was performed at a 0.5 mL/min flow rate and at 45 °C. The mobile phase was 1 mM H_2_SO_4_. The glucan conversion was calculated according to the follow equation:

$${\text{Glucan conversion}} = \frac{Cg \times Vg}{M \times W} \times 100,$$

where *Cg* is the obtained glucose concentration (g/L); *Vg* is the volume of enzymatic hydrolysis (L); *M* is the amount of dry SPRs (g); and *W* is the maximum amount of glucose released from dry SPRs (%). Three independent replicates were performed. Two-tailed Student’s t tests were used for statistical analysis, and p < 0.05 was considered statistically significant.

### Ethanol production after enzymatic hydrolysis

Cellulase from *T*. *reesei* TX, commercial pectinase and their mixtures were added to SPRs. The final substrate concentrations were adjusted to 36 % by supplementing citric acid buffer (10 mM, pH 4.8). All the tubes were incubated in an oscillating machine at 45 °C and 200 rpm for 6 h. Subsequently, 53 units of α-amylase were added and incubated at 90 °C for 2 h. Next, the reactions were cooled to 60 °C with the addition of 34 units of α-glucosidase. The reaction systems were incubated for 6 h to obtain the maximum glucose release. Approximately 1.4 g of high sugar tolerant instant dry yeast (*S. cerevisiae*; Sunkeen, China) was dissolved in 50 mL of solution containing 2 % glucose at 30 °C for 1 h. Then, 2 mL of the solution was inoculated into 30 mL of the SPR enzymatic hydrolysates. The fermentations were incubated at 30 °C for 72 h. The HPLC system (Hitachi, Tokyo, Japan) equipped with an Aminex HPX-87H column (7.8 × 300 mm, 9 µm particle size, Bio-Rad Laboratories, Hercules, CA, USA) and an RI detector (Model L-2490, Hitachi, Tokyo, Japan) were used for glucose and ethanol measurement. The separation was performed at a 0.5 mL/min flow rate and at 45 °C. The mobile phase was 1 mM H_2_SO_4_.

The ethanol yield and glucose conversion were calculated according to the following equations:

$${\text{Ethanol yield}} = \frac{Ce \times V}{M} \times 100$$

and

$${\text{Glucose to ethanol conversion}} = \frac{Ce \times V}{M \times W \times 0.51} \times 100,$$

where *Ce* is the ethanol concentration (g/L); *V* is the fermentation volume (L); *M* is the amount of dry SPRs (g); and *W* is the maximum amount of glucose released from dry SPRs (%). Three independent replicates were performed.

## Additional file

10.1186/s13068-016-0464-7 The change of glucan conversions of high-gravity SPRs when commercial pectinase solutions were added. All the reactions were performed at 45 °C and 200 rpm for 6 h with an initial pH of 4.8. The concentration of SPRs in reaction system was 36 % (w/v). Three independent replicates were carried out.

## Abbreviations

SPRs: sweet potato resides
FPase: filter paperase
FPU: filter paperase unit
PGU: polygalacturonase unit
