# Supplementary material for: An environmentally friendly and productive process for bioethanol production from potato waste
Source: Biotechnol Biofuels. 2016 Mar 2;9:50. doi: 10.1186/s13068-016-0464-7 (PMC4776430; doi:10.1186/s13068-016-0464-7)
Supplement: Supplementary file 1 — 10.1186/s13068-016-0464-7 The change of glucan conversions of high-gravity SPRs when commercial pectinase solutions were added. All the reactions were performed at 45 °C and 200 rpm for 6 h with an initial pH of 4.8. The concentration of SPRs in reaction system was 36 % (w/v). Three independent replicates were carried out. [file 13068_2016_464_MOESM1_ESM.docx]

**Table S1 The change of glucan conversions of high-gravity SPRs when commercial pectinase solutions were added.** All the reactions were performed at 45 °C and 200 rpm for 6 hours with an initial pH of 4.8. The concentration of SPRs in reaction system was 36% (w/v). Three independent replicates were carried out.

| The loading amount of  pectinase  (PGU/g dry SPRs) | 0 | 1000 | 5000 | 10000 | 20000 |
| --- | --- | --- | --- | --- | --- |
| Glucan Conversion (%) | 33.47 ± 2.18 | 35.61 ± 5.45 | 37.75 ± 1.60 | 40.93 ± 1.66 | 39.03 ± 1.00 |
